# *Stephania tetrandra S.* Moore: a promising candidate drug for treating diabetic kidney disease

**DOI:** 10.3389/fphar.2025.1651023

**Published:** 2025-09-15

**Authors:** Wenru Wang, Jixin Li, Yan Yan, Qin Zeng, Lei Yan, Xinhui Wang, Ying Liang, Renhuan Yu

**Affiliations:** ^1^ Department of Nephrology, Xiyuan Hospital of China Academy of Chinese Medical Sciences, Beijing, China; ^2^ Hubei University of Chinese Medicine, Wuhan, China; ^3^ Zhujiang Hospital, Southern Medical University, Guangzhou, China

**Keywords:** diabetic kidney disease, *Stephania tetrandra S.* Moore, active compounds, Chinese herbal medicine, mechanism

## Abstract

Diabetic kidney disease (DKD) is a common microvascular complication of diabetes. With the continuous rise in the prevalence of diabetes, it has become the primary cause of end-stage renal disease. Currently, there are no effective clinical treatments available to reverse the progression of DKD. *Stephania tetrandra S.* Moore, a traditional Chinese medicine, has demonstrated significant value in the prevention and treatment of DKD due to its active components. This study focuses on exploring the molecular mechanisms through which the primary active components of *S. tetrandra*, tetrandrine/sinomenine and fangchinoline, exert renal protective effects via multiple pathways, including regulating inflammatory responses, antagonizing oxidative stress, improving glomerular endothelial function, modulating podocyte damage, and intervening in lipid metabolism disorders. These findings provide a theoretical basis for the development of novel therapeutic agents for DKD.

## Introduction

Diabetic kidney disease (DKD) is the leading cause of chronic kidney disease and end-stage renal disease worldwide. Its typical clinical features include persistent albuminuria and progressive renal function decline (Liu et al., 2024; Liu et al., 2023). The pathophysiological process of DKD is highly complex, primarily driven by a hyperglycemic environment, involving interactions between genetic susceptibility and environmental factors that collectively trigger and promote the onset and progression of kidney damage (Mora-Fernandez et al., 2014; Liu et al., 2020; Ma et al., 2018; Ma et al., 2024). High glucose toxicity is the core initiating factor of this disease. It first induces dysfunction of glomerular podocytes and endothelial cells, triggering a series of cascading reactions, including local infiltration of inflammatory mediators, activation of oxidative stress mediated by reactive oxygen species (ROS), and abnormal deposition of the extracellular matrix (ECM), ultimately leading to thickening of the glomerular basement membrane. Concurrently, disrupted glomerular hemodynamics exacerbates the damage process and may progress to characteristic nodular glomerulosclerosis (Kanwar et al., 2011). In addition, the damaging effects of hyperglycemia also significantly affect the renal tubulointerstitial tissue, resulting in renal tubule hypertrophy, thickening of the renal tubule basement membrane, and undergoing epithelial-mesenchymal transition (EMT), a key pathological process that promotes fibrosis (Zhang et al., 2021; Zhao et al., 2024). A meta-analysis (Tziastoudi et al., 2020) showed that the development of DKD is associated with at least 66 genetic polymorphisms. These genetic variations are widely involved in multiple signaling pathways related to pathogenic mechanisms, such as inflammatory responses, oxidative stress, endothelial dysfunction, and lipid metabolism disorders.

In view of the complexity and networked nature of the pathogenesis of DKD, traditional Chinese medicine (TCM), with its unique advantages of multi-target and multi-pathway synergistic regulation, has demonstrated significant therapeutic potential in regulating the pathological process of DKD (Lin et al., 2022; Liu et al., 2018; Geng et al., 2023). In the DKD-related treatment guidelines and expert consensus published in China, various single Chinese herbal medicines and compound preparations have been explicitly listed as recommended treatment options (Chung et al., 2023). *Stephania tetrandra S.* Moore, a traditional Chinese medicine, is derived from the dried roots of the Stephaniaceae plant *S. tetrandra*. It has a bitter and cold nature and is used to dispel wind-dampness, promote the flow of qi and blood, and promote diuresis and reduce swelling. Due to its diuretic properties, *S. tetrandra* is widely used in TCM formulas for the treatment of chronic kidney disease (including DKD) (Cui et al., 2023; Wang et al., 2024; Liu et al., 2025). Modern pharmacological research has shown that *S. tetrandra* contains a wealth of active ingredients. To date, at least 48 active substances, including alkaloids, flavonoids, and steroidal compounds, have been isolated and identified from it (Zhang et al., 2020). Among these, biphenylisoquinoline alkaloids (BBIQs), particularly tetrandrine/sinomenine and fangchinoline, have been identified as the primary pharmacologically active components, It was found that tetrandrine/sinomenine and fangchinoline amounted to 1.2977%–1.9887% and 0.7437%–0.8973% in *S. tetrandra* (Tao et al., 2022) and the quality control index components of *S. tetrandra* in pharmacopoeia standards, according to the Chinese pharmacopeia (2015 edition), the total fraction of tetrandrine and fangchinoline in *S. tetrandra* should not be less than 1.6%. Numerous studies have demonstrated that these alkaloids exhibit a wide range of pharmacological activities, including anti-inflammatory (Wang et al., 2025), antioxidant (Li et al., 2024), anti-fibrotic (Zhuo et al., 2025), and anti-tumor (Song et al., 2025) effects, and show potential in rheumatoid arthritis and pulmonary fibrosis, and cancer treatment studies (Li J. M. et al., 2023; Chu et al., 2024; Xie et al., 2025).

Currently, research into the pharmacological basis of active components in medicinal plants and their molecular mechanisms of action has become a focal point of academic attention. However, the precise molecular mechanisms and specific targets of the key active components in TCM *S. tetrandra* in intervening in DKD remain poorly understood. In light of this, this study aims to thoroughly elucidate the potential molecular mechanisms and action targets of tetrandrine/sinomenine and fangchinoline in intervening in DKD, with the goal of clarifying the biological basis of their renal protective effects. This research seeks to provide important scientific evidence and potential new intervention strategies for expanding the clinical application of *S. tetrandra* and its active components in the treatment of DKD.

### Mechanisms of action of tetrandrine/sinomenine and fangchinoline in DKD

**FIGURE 1:**
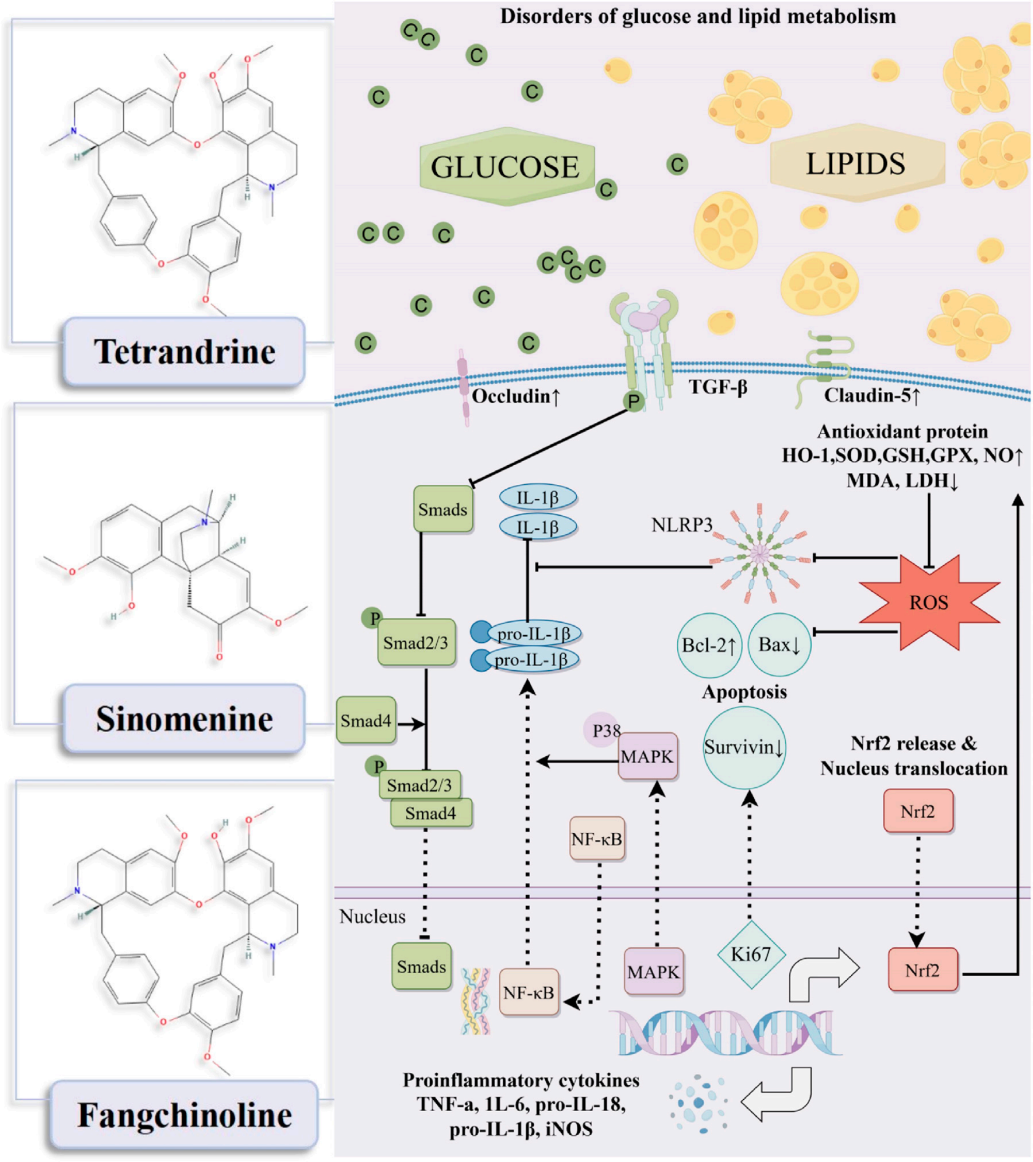
Mechanism diagram of tetrandrine/sinomenine and fangchinoline acting on diabetic kidney disease.

The mechanisms by which tetrandrine/sinomenine and fangchinoline intervene in DKD are primarily manifested in anti-inflammatory, antioxidant stress, anti-fibrotic, repair of glomerular endothelial damage, reduction of cell apoptosis, and regulation of lipid metabolism, thereby synergistically regulating the core pathological processes of DKD through multiple pathways (Table 1; Figure 1).

**TABLE 1:** Mechanisms of action of tetrandrine/sinomenine and fangchinoline on diabetic kidney disease.

Compound name	Model	Dosages	Time	*In Vivo/In Vitro*	Effect	Mechanism	Specific pathways	References
tetrandrine (sinomenine)	STZ-induced diabetes rats	10,20,40 mg/kg	6W	*In Vivo*	↓FBGLs, TC, TG, kidney/body ratio, Cr, BUN, UA, β2-MG	anti-inflammatory, anti-oxidative stress, attenuates apoptosis and fibrosis	↓JAK2/STAT3 ↑SOCS1 signaling pathway	Zhu et al. (2021)
	H_2_O_2_ induced HK-2 cells	20,80,320 μg/mL	24 h	*In Vitro*	—	anti-oxidative stress	↓ROS ↑GPX1/SOD2	
	STZ-induced diabetes rats	20,40 mg/kg	8W	*In Vivo*	↓24hUTP	anti-inflammatory,regulate lipid metabolism	↓AGEs/RAGE, IL-17, JAK, TNF signaling pathway	Li et al. (2023b)
	STZ-induced diabetes rats	20,40 mg/kg	6W	*In Vivo*	↓GLU, UAE	anti-inflammatory,amelioration of glomerular endothelial injury	↑C/EBP-α/claudin-5 signaling pathway	Zhang and Wang (2022)
	HG-induced HrGECs	50,100 μM	24 h	*In Vitro*	—			
	STZ-induced diabetes rats	5,15,30 mg/kg	8W	*In Vivo*	↓GLU, Cr, proteinuria, UREA	anti-inflammatory, anti-oxidative stress, attenuates apoptosis	↑Nrf2/HO-1 signaling pathway	Su et al. (2020)
	HG-induced HrGECs	20 μg/mL	24 h	*In Vitro*	—	anti-oxidative stress, amelioration of glomerular endothelial injury	↓RhoA/ROCK signaling pathway	Yin et al. (2016)
fangchinoline	STZ-induced diabetes rats	3 mg/kg	8W	*In Vivo*	↓GLU, Cr, ALB, UREA, hs-CRP	anti-inflammatory,anti-oxidative stress	↓MAPK signaling pathway	Jiang et al. (2020)

### Anti-inflammatory

Systemic inflammatory response is a key pathological mechanism driving the progression of DKD (Bai et al., 2024). Research has confirmed that neutrophil extracellular traps (NETs) induce characteristic sterile inflammation in DKD by activating NLRP3 inflammasomes in glomerular endothelial cells in both human and mouse models (Wang et al., 2023), laying the theoretical foundation for targeting inflammation as a treatment for DKD (Rayego-Mateos et al., 2023). Experimental evidence shows that sinomenine can reversibly reverse the upward trend of ICAM-1 and interleukin (IL)-6 in a dose-dependent manner, and notably, chronic 6-week administration significantly ameliorated elevated fasting blood glucose levels (FBGLs) in STZ-induced diabetes rats in a dose-dependent manner, while also improving other metabolic and renal function parameters without significant hepatotoxicity (Zhu et al., 2021). In STZ-induced diabetes rats, 6-week sinomenine treatment significantly ameliorated elevated blood glucose (GLU) levels and reduced urinary albumin excretion (UAE) values, meanwhile, further investigation revealed that its anti-DKD mechanism may play a protective role in the kidney by down-regulating the expression of IL-18/IL-1β in human glomerular endothelial cells (HrGECs) and renal tissues (Zhang and Wang, 2022). In addition, tetrandrine significantly downregulated GLU, serum creatinine (Cr), proteinuria, and urea nitrogen levels in STZ-induced diabetes rats, and significantly decreased serum levels of IL-6, tumor necrosis factor (TNF)-α, and inducible nitric oxide synthase, while increasing levels of the anti-inflammatory factor IL-10. (Su et al., 2020). Fangchinoline, another major active component of *S. tetrandra*, effectively downregulates IL-6 and TNF-α levels by inhibiting p38 MAPK pathway activation, while improving the levels of GLU, Cr, ALB in STZ-induced diabetes rats (Jiang et al., 2020).

### Antioxidant stress

Oxidative stress is the core pathological hub of DKD, characterized by a cascade reaction triggered by an imbalance in the oxidative/antioxidant system. This imbalance activates downstream signaling pathways, exacerbating inflammation, autophagy dysfunction, and fibrosis, ultimately accelerating structural and functional damage to the kidneys (Chen et al., 2023). Key experimental evidence shows that sinomenine significantly improves high glucose-induced oxidative damage in the HK-2 cell model, as evidenced by increased cell viability, while upregulating glutathione peroxidase 1 (GPX1), superoxide dismutase 2 (SOD2), and reduced glutathione (GSH) levels, and reducing ROS production (Zhu et al., 2021). In addition, tetrandrine effectively reduced malondialdehyde (MDA), lactate dehydrogenase (LDH), and ROS levels in STZ-induced DKD rats by activating the nuclear factor erythroid 2-related factor 2 (Nrf2)/heme oxygenase-1 (HO-1) signaling axis, while increasing SOD activity (Su et al., 2020). This mechanism is consistent with the core regulatory role of Nrf2, by inducing the expression of antioxidant genes such as HO-1, it alleviates oxidative stress damage in a high-sugar environment (Wang et al., 2015). Fangchinoline reverses the increase in MDA and decrease in nitric oxide (NO) in rat kidney tissue, while restoring SOD activity (Jiang et al., 2020). It is worth noting that sinomenine also regulates the RhoA/ROCK pathway through Nrf2-mediated ROS inhibition, correcting the oxidative microenvironment induced by high glucose levels (Yin et al., 2016), highlighting the importance of networked regulation of antioxidant pathways.

### Repair of glomerular endothelial damage

Hyperglycemia-induced endothelial cell dysfunction in the glomerulus is a key factor in the development of proteinuria in DKD. It directly damages the endothelial barrier and disrupts the paracrine communication between endothelial cells and podocytes, leading to an abnormal increase in the permeability of the glomerular filtration barrier (Chen et al., 2024). Research has confirmed that in DKD rat models and HrGECs stimulated with high glucose, sinomenine can enhance tight junction integrity by upregulating the C/EBP-α/claudin-5 signaling axis (Zhang and Wang, 2022). After 24 h of drug intervention, the RhoA/ROCK pathway activation was effectively inhibited, while endothelial permeability was reduced and the expression of tight junction protein occludin was increased (Yin et al., 2016). This multi-pathway synergistic action jointly maintains the structural and functional integrity of the glomerular filtration barrier, providing a mechanistic basis for sinomenine to alleviate proteinuria in DKD.

### Anti-fibrotic

Although research on DKD has traditionally focused on glomerular damage, the key role of tubulointerstitial lesions in disease progression is becoming increasingly apparent (Jia et al., 2024; Ma et al., 2023). Renal interstitial fibrosis, characterized by damage to renal parenchymal cells and massive ECM deposition, is the core driver of late-stage renal function deterioration in DKD (Zhou et al., 2024). In this process, EMT has been identified as a key mechanism by which renal tubular epithelial cells lose their polarity and acquire a mesenchymal phenotype (Gilbert and Cooper, 1999), yet there has been a long-standing lack of effective intervention strategies. Previous studies have reported that sinomenine can effectively improve EMT-related proteins α-SMA, E-cadherin, fibronectin, and ECM deposition by blocking TGF-β/Smad3 and Wnt/β-catenin signal transduction (Qin et al., 2016). In the DKD rat model, dose-dependent reversal of abnormal overexpression of TGF-β1, type I collagen (Collagen-1), and fibronectin (Zhu et al., 2021). The latest multi-omics study further reveals that sinomenine significantly reduces albuminuria levels in STZ-induced DKD rats by regulating differentially expressed gene networks (especially those enriched in inflammation and EMT pathways), providing multidimensional evidence for delaying the progression of DKD (Li Y. et al., 2023).

### Regulate of lipid metabolism

The role of lipid toxicity in kidney disease was first proposed by Moorhead et al., in 1982 and further updated by Ruan et al., confirming that lipid metabolism disorders (manifested as dyslipidemia and tissue lipid accumulation) are key factors driving the progression of various kidney diseases, including DKD (Zhang et al., 2025; Zhu et al., 2023). Among these, abnormal lipid accumulation in renal podocytes has been identified as the core pathological mechanism underlying the development of DKD (Chen et al., 2017). This process triggers pathological activation of ROS production, which in turn triggers a vicious cycle of oxidative stress, inflammatory cascade reactions, and cell death signaling pathways, ultimately accelerating renal function damage (Mitrofanova et al., 2021). Recent metabolomics research has revealed that sinomenine can systematically regulate lipid metabolism disorders in DKD, specifically through the synergistic regulation of three key pathways: linoleic acid metabolism, arachidonic acid metabolism, and glycerophospholipid metabolism (Li J. M. et al., 2023). This provides new evidence for the development of targeted treatment strategies for lipotoxicity.

### Reduce of cell apoptosis

Proteinuria, as the core clinical manifestation of DKD, mainly originates from podocyte apoptosis, detachment, and structural damage. In the cell apoptosis regulatory network, the dynamic balance between pro-apoptotic protein Bax and anti-apoptotic protein Bcl-2 is crucial. Studies have shown that 6 weeks of sinomenine administration significantly downregulates Bax expression and upregulates Bcl-2 levels in the kidneys of DKD rats, effectively antagonizing podocyte apoptotic damage (Zhu et al., 2021). Tetrandrine can activate the cell proliferation marker Ki67 and the apoptosis inhibitory protein survivin, while significantly reducing the Bax/Bcl-2 ratio (Su et al., 2020). This synergistic regulation of the apoptosis signaling pathway provides a key molecular basis for alleviating DKD proteinuria.

## Conclusion and perspectives

In summary, existing studies have gradually revealed the multidimensional pharmacological activities of tetrandrine/sinomenine and fangchinoline in the prevention and treatment of DKD. Their core mechanisms involve significantly inhibiting inflammatory responses, effectively antagonizing oxidative stress damage, and improving the structure and function of glomerular endothelial cells.

However, current evidence is mainly derived from preclinical studies, and its translation to the clinic faces key challenges, including the potential toxicity of the active ingredients, bioavailability limitations, and the lack of support from high-quality clinical data. Future research should prioritize rigorous clinical trials to validate their efficacy and safety, explore pharmacokinetic properties and toxicity mechanisms to optimize therapeutic strategies. Furthermore, mechanistic studies on the effects of *S. tetrandra* active components in intervening DKD remain relatively scarce and lack systematic depth. To address this, future research prospects could focus on deepening the understanding of its multi-target synergistic effects; and, on this basis, working on the development of more potent active compounds and multi-target synergistic-based therapeutic strategies. These efforts will lay a solid foundation for elucidating the scientific connotation of *S. tetrandra* in the treatment of DKD and promoting its modernization research.
